# NPLOC4 Inhibition Remodels Tumor Microenvironment via M2-to-M1 Macrophage Reprogramming and Boosts Anti-PD-1 Response in Liver Cancer

**DOI:** 10.7150/ijbs.125201

**Published:** 2026-02-11

**Authors:** Xingxing Gao, Hechen Huang, Caixu Pan, Jiacheng Huang, Junru Chen, Shengyong Yin, Lin Zhou, Shusen Zheng

**Affiliations:** 1 Division of Hepatobiliary and Pancreatic Surgery, Department of Surgery, The First Affiliated Hospital, Zhejiang University School of Medicine, Hangzhou, 310003, China.; 2 Department of Thyroid Surgery, The First Affiliated Hospital, Zhejiang University School of Medicine, Hangzhou, China.; 3 NHC Key Laboratory of Combined Multi-organ Transplantation, Key Laboratory of Organ Transplantation, Zhejiang Province, China.; 4 Institute of Organ Transplantation, Zhejiang University, China.

**Keywords:** NPLOC4, macrophage, immunotherapy, tumor microenvironment, hepatocellular carcinoma

## Abstract

The PD-1/PD-L1 axis represents a well-established immunotherapeutic target. Nevertheless, anti-PD-1/PD-L1 therapeutics have shown limited efficacy in the management of solid tumors, particularly in the context of hepatocellular carcinoma (HCC). Among the various factors contributing to the resistance to anti-PD-1/PD-L1 therapy, tumor-associated macrophages (TAMs) have attracted significant interest because of the immunosuppressive properties. NPLOC4 has been explored as an antitumor drug target. However, whether NPLOC4 functions in TAMs or immunotherapy is unclear. Here, we report a new role for NPLOC4^+^ TAMs in inhibiting antitumor immune responses by facilitating the proteasomal degradation of RIG-I. Clinical specimens revealed that the number of NPLOC4^+^ TAMs are negatively correlated with the prognosis of patients with HCC. Proteomic data and *in vitro*/*in vivo* experiments demonstrated that NPLOC4 inhibits the type I interferon pathway in TAMs, promotes M2 polarization, and suppresses CD8^+^ T-cell infiltration, thereby creating an immunosuppressive microenvironment in HCC. NPLOC4 can bind to RIG-I and mediate its ubiquitination-mediated degradation, thus suppressing the type I interferon pathway. Animal studies have indicated that disulfiram/copper (DSF/Cu) can target the NPLOC4 protein, and that the combination of DSF/Cu with PD-1 therapy significantly inhibits HCC growth. In conclusion, targeting NPLOC4^+^ TAMs can significantly increase the resistance of HCC to anti-PD-1 therapy, which makes it a promising novel immune target for HCC treatment.

## Introduction

In 2020, primary liver cancer ranked as the third most common cause of cancer-related mortality globally, with hepatocellular carcinoma (HCC) accounting for 85% of all cases 1. Currently, radical surgery remains the main treatment for liver cancer. Nonetheless, the stringent surgical criteria imply that the majority of patients with liver cancer patients can only undergo alternative conservative interventions, including transarterial chemoembolization (TACE), local ablation, and systemic combined therapy 2. In recent years, immune checkpoint blockade (ICB) therapy targeting programmed cell death protein-1 (PD-1) and its ligand (PD-L1) has made significant progress in cancer management. By enhancing the tumor-killing capacity of T cells, this approach has been approved for treating advanced hepatocellular carcinoma and has demonstrated notable therapeutic advantages 3. However, the objective response rate to ICB therapy in patients with advanced liver cancer is only 16-20%, and many tumors that show a significant initial response to treatment often progress into drug-resistant malignant tumors 4.

Growing evidence suggests that the tumor microenvironment (TME) is pivotal in mediating resistance against cancer immunotherapies 5. Among the diverse immune cell populations that infiltrate cancerous tissues, macrophages predominate in number and play a central role in modulating the TME 6. Traditionally, tumor-associated macrophages (TAMs) are broadly classified into two subtypes: M1 (proinflammatory, tumor-killing) and M2 (anti-inflammatory, tumor-promoting) 7. In the tumor microenvironment of hepatocellular carcinoma, most TAMs polarize into the M2 phenotype, contributing to the establishment of an immunosuppressive microenvironment 8. TAMs actively facilitate promote tumor metastasis and angiogenesis, simultaneously suppressing the antitumor immunity 9. Emerging studies have indicated that TAMs exhibit dual regulatory effects on the antitumor activity of chemotherapy, radiation therapy, antiangiogenic agents, and immunotherapies. These opposing functions are closely related to their heterogeneity 10.

NPL4 homolog, ubiquitin recognition factor (NPLOC4) is a cofactor of the p97 protein and has been shown to participate in a wide range of independent cellular processes, including endoplasmic reticulum (ER)- and mitochondria-associated degradation 11-12. With the help of NPLOC4, p97 is recruited to ubiquitinated substrates to extract its target proteins from their cellular environment, which is primarily leads to proteasomal degradation 13. Early studies have shown that interfering with the expression of NPLOC4 in tumor cells inhibits tumor progression by affecting cell proliferation and apoptosis in melanoma, kidney chromophobe, and glioma 14-15. In recent years, NPLOC4 has also been reported to be associated with the formation of an immunosuppressive tumor microenvironment. Blocking the p97-Npl4 interaction inhibited the development of tumor-infiltrating regulatory T cells (Tregs) and enhanced tumor immunity 16. A genome-wide CRISPR-based screening study also revealed that knockout of NPLOC4 in tumor cells can increase immune signaling in gliomas and improve the sensitivity to adoptive immunotherapy 17. However, the expression of NPLOC4 in TAMs and whether it is involved in regulating the tumor microenvironment remain unclear. As a solid tumor characterized by a high macrophage density, the involvement of NPLOC4 in the development and progression of HCC merits further exploration.

In this study, we identified a notable inverse correlation between the presence of NPLOC4^+^ TAMs in the tumor microenvironment and the prognosis of HCC patients. We demonstrated that NPLOC4 promotes M2 polarization of TAMs, thereby impairing the anti-tumor response of CD8^+^ T cells. Mechanistically, NPLOC4 mediates the ubiquitin-mediated degradation of RIG-I, inhibits type I interferon secretion, and impairs the antitumor function of macrophages. Finally, targeting NPLOC4^+^ TAMs promotes the M1 reprogramming of TAMs, enhances the killing function of CD8^+^ T cells, and ultimately significantly potentiates the therapeutic effect of anti-PD-1 treatment. Our results demonstrate that NPLOC4 serves as a prognostic biomarker, and targeting NPLOC4 in TAMs improves the effectiveness of anti-PD1 therapy, offering a potential combinatorial approach for HCC.

## Materials and Methods

### Clinical tissues collection

We collected 32 tissue samples in total from patients with HCC who had curative resection surgery at our hospital between 2013 and 2014. The tissue samples underwent immunofluorescence staining to visualize cellular markers, followed by survival analyses correlating with patient outcomes. This study was approved by the Ethics Committee of our center (20231019). The clinical characteristics of all patients were summarized in Table S1.

### Cell lines

THP-1 cell lines was obtained from America Type Culture Collection (Manassas, VA) in 2017. HEP-53.4 was obtained from Cell Line Service GmbH in 2023. The Cell line authentication was conducted at the same time.

### Cell culture and transfection

The supplementary materials illustrate the methodologies for cell culture and transfection.

### Analysis of public sc-RNA datasets

The human HCC scRNA-seq datasets were downloaded from GEO database (GSE202642 and GSE242889) 18-19. The expression of NPLOC4 in macrophages were analyzed. Supplementary Materials shows the specific details of data processing, dimension reduction and clustering of the scRNA-seq data.

### Generation and intervention of murine tumor models

In line with the protocols of the Institutional Animal Care and Use Committee, all animal experiments were approved by the Animal Care Committee of our hospital (2024-69). C57BL/6J male mice aged 6-8 weeks were primarily utilized in this study. Nploc4^flox^ mice (The Cyagen Biosciences) were crossed with Lyz2^Cre^ mice (The Cyagen Biosciences) to generate Nploc4^cKO^ mice. The experimental mice were housed in the Specific Pathogen-Free (SPF) animal facility of our institution. Subsequent to the establishment of the murine tumor model, a comprehensive account of the drug treatment regimens is provided in the supplementary materials.

### Flow cytometry

Mononuclear cells were isolated from single-cell cultures via OptiPrep Density Gradient Medium. Post-antibody incubation, multicolor flow cytometry was applied to detect CD8^+^ T cells and macrophages. To evaluate cytotoxic activity, CD8^+^ T cells were stimulated with Leukocyte Activation Cocktail (550583, BD Pharmingen, San Diego, CA, USA) for 5 hours. The Supplementary Materials detail the specific staining protocols and antibodies employed.

### Immunofluorescence, and immunoblotting assay

Immunofluorescence staining was conducted on 3-mm tissue slides, and the acquired images were independently evaluated by two pathologists. The detailed protocols are described in the Supplementary Materials.

### RNA extraction and real time qPCR (RT-qPCR)

Total RNA was extracted using RNA-Quick Purification Kit (Esunbio). Total RNA was reverse transcribed into cDNA with HiScript II Q Select RT SuperMix for qPCR (R233-01, Vazyme) and HiScript III All-in-one RT SuperMix Perfect for qPCR (R333-01, Vazyme). RT-qPCR was performed with ChamQ SYBR Green Master Mix (Vazyme) on QuantStudio 5 Real-Time PCR System (ThermoFisher). The relative cycle threshold (CT) value was computed with 2^ΔΔCt^ and normalized to that of ACTB. The primers of all genes were ordered from Tsingke Biological Technology and listed in Table S3.

### Functional enrichment analysis

GO and KEGG pathway enrichment analyses were utilized to assess the major GO terms (biological processes, molecular functions, and cellular components) and KEGG pathways linked to the DEGs. This analysis was conducted via the “clusterProfiler” package in R software 20.

### Proteomics sequencing

Total proteins were extracted from the THP-1 cells that transfected with si-NPLOC4 and negative control (NC), respectively. Triplicate samples were assigned in each group and the knockdown efficiency was prechecked by qPCR. The targeted proteomics procedures are detailed in the Supplementary Materials.

### Statistical analysis

To compare variables following a normal distribution, Student's t-test was applied, whereas the Wilcoxon rank-sum test was utilized for groups with non-normal distributions. Survival analysis was conducted using the survival and survminer packages in R, with group differences evaluated via the log-rank test. All experiments were performed in triplicate, and statistical analyses were carried out using R software (version 3.6.3).

## Results

### NPLOC4 was upregulated in tumor-associated macrophages

**Figure 1:**
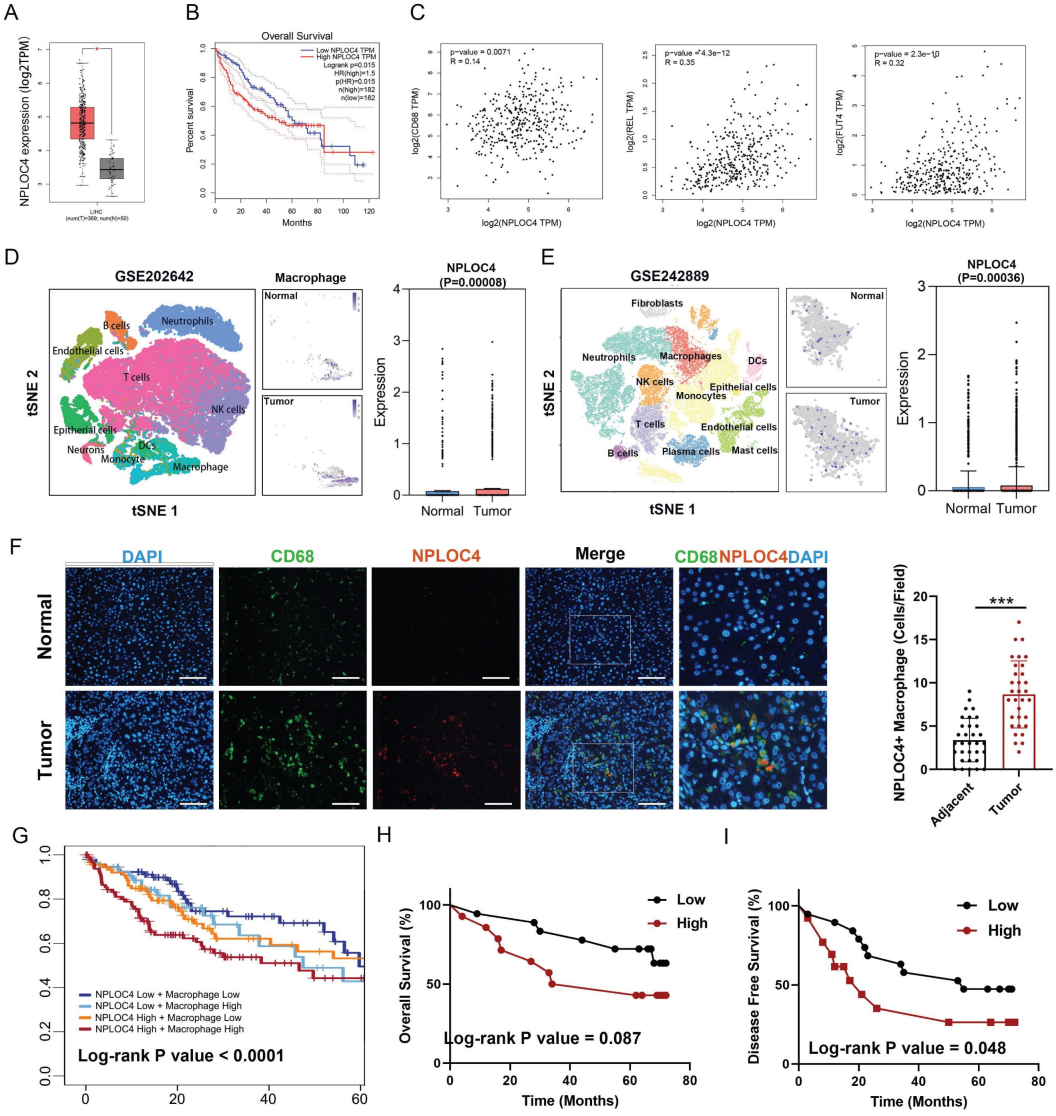
** NPLOC4 is up-regulated on macrophages in the HCC microenvironment and associated with poor prognosis.** (A) Boxplots showing the NPLOC4 expression in HCC and corresponding normal tissues from RNA-seq results of the TCGA database. (B) Association of NPLOC4 expression with prognosis of HCC patients in TCGA database. (C) The correlation of NPLOC4 expression with myeloid-related genes in HCC. (D-E) NPLOC4 expression in tumor-infiltrating macrophages and corresponding normal tissues from single-cell RNA-seq datasets of HCC. (F) Representative images of NPLOC4 (red), CD68 (green), and DAPI (blue) immunofluorescence staining in HCC tissues at × 400 magnification. According to the average number of NPLOC4^+^ TAMs in 10 random fields of HCC tissues and clinical case data, the relationship between NPLOC4^+^ TAM relative expression in tumor tissues and adjacent tissues was analyzed. Data are expressed as mean ± SEM (n = 32). Scale bar: 100 μm. (G) The 5-year survival curves of high and low NPLOC4 or macrophages groups in the TCGA database were compared. (H-I) The total survival (H) and disease-free survival (I) curves of the high (n=16) and low (n=16) NPLOC4^+^ TAMs group were compared. n: number of biological replicates. *p < 0.05; **p < 0.01; ***p < 0.001; ****p < 0.0001. Abbreviations: DAPI 4′,6-diamidino-2-phenylindole.

We first screened the expression of NPLOC4 in tumor and normal tissues in the TCGA database 21. NPLOC4 mRNA expression was notably upregulated in 7 out of 23 tumor tissue types, encompassing hepatocellular carcinoma (LIHC), cholangiocarcinoma (CHOL), stomach adenocarcinoma (STAD), lung adenocarcinoma (LUAD), esophageal carcinoma (ESCA) and lung squamous cell carcinoma (LUSC) (Figure 1A, Figure S1A). Prognostic analysis revealed that elevated NPLOC4 expression was linked to unfavorable outcomes in various cancer types, such as HCC, adrenocortical carcinoma (ACC) and skin cutaneous melanoma (SKCM) (Figure 1B, Figure S1B). By employing the ESTIMATE algorithm for estimating stromal and immune cell components in malignant tumor tissues, we identified an inverse correlation between NPLOC4 expression and immune scores in HCC (Figure S1C) 22. NPLOC4 expression exhibited a positive correlation with the expression of myeloid-associated genes, such as CD68, REL, FUT4, and immune checkpoint molecules, such as PD-L1 and CTLA4 (Figure 1C, Figure S1D). Utilizing the TIMER 2.0 website, we discovered that NPLOC4 expression also showed a positive association with macrophage infiltration (Figure S1E) 23. Analysis of publicly available scRNA-seq datasets revealed elevated NPLOC4 expression in TAMs compared with that in macrophages in adjacent tissues of patients with HCC. (Figure 1D-E). Immunofluorescence staining of in tumor and adjacent tissues from patients with HCC revealed that NPLOC4 expression was upregulated in TAMs (Figure 1F). Prognostic analysis also revealed that high NPLOC4 expression and TAM infiltration were associated with poor prognosis in patients with HCC in the TCGA dataset (n=365), CHCC-HBV dataset (n=159) and our center (n=32) (Figure 1G-I, Figure S1F). These results suggested that NPLOC4 was upregulated in TAMs.

### NPLOC4^+^ TAMs promoted HCC progression through the inhibition of CD8^+^ T cell infiltration and the induction of their dysfunction

**Figure 2:**
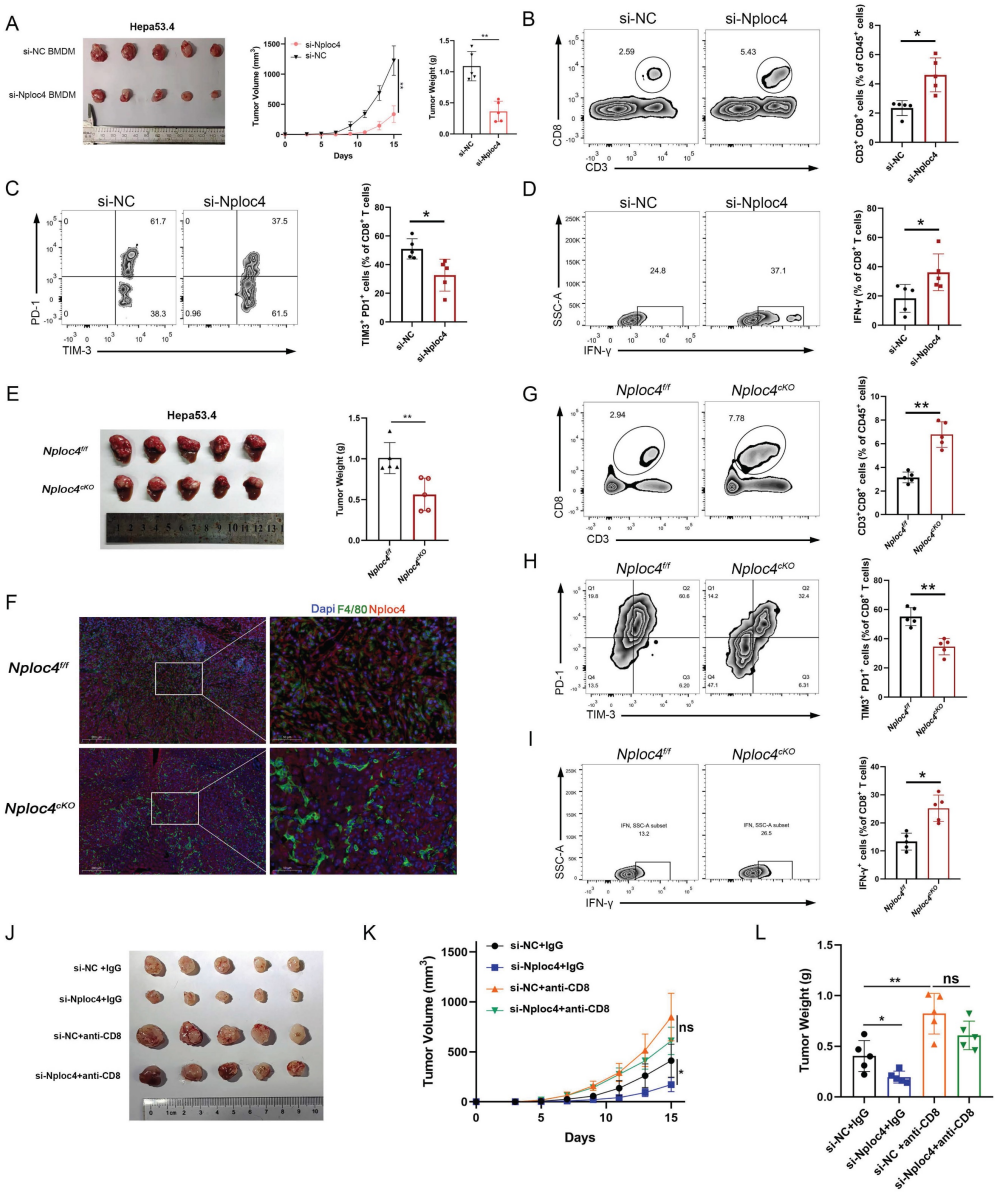
** NPLOC4^+^ TAMs mediate hepatocellular carcinoma progression by inhibiting CD8^+^ T cells infiltration and cytotoxic function.** (A) A tumor model was established by subcutaneous inoculating HEP53.4 mixed with macrophages (transfected with si-NC or si-NPLOC4, at a 2:1 ratio) into C57BL/6 mice. tumor growth was monitored regularly, and the data of tumor volume was represented as mean ± SEM (n = 5 in each group). (B) Flow cytometry of CD8^+^ T cell ratio to CD45^+^ in NPLOC4 knockdown and control groups was expressed as mean ± SEM (n = 5 in each group). (C) Flow cytometry of the exhausted status of CD8^+^ T cells (PD-1^+^ and TIM-3^+^ labeling) in NPLOC4 knockdown and control groups was expressed as mean ± SEM (n = 5 in each group). (D) Flow cytometry of the cytotoxic function of CD8^+^ T cells (IFN-γ labeling) in NPLOC4 knockdown and control groups was expressed as mean ± SEM (n = 5 in each group). (E) The diagram and tumor weight of tumor growth in orthotopic tumor model inoculating HEP53.4 cells in Nploc4^f/f^ and Nploc4^cKO^ groups. (F) Representative images of immunofluorescence staining in orthotopic tumor tissues of the Nploc4^f/f^ and Nploc4^cKO^ groups. (G) Flow cytometry of CD8^+^ T cell ratio to CD45^+^ in Nploc4^f/f^ and Nploc4^cKO^ groups (n = 5 in each group). (H) Flow cytometry of the exhausted status of CD8^+^ T cells (PD-1^+^ and TIM-3^+^ labeling) in Nploc4^f/f^ and Nploc4^cKO^ groups (n = 5 in each group). (I) Flow cytometry of the cytotoxic function of CD8^+^ T cells (IFN-γ labeling) in Nploc4^f/f^ and Nploc4^cKO^ groups (n = 5 in each group). (J-L) The diagram (J), tumor volume (K) and weight (L) of tumor growth in mouse model inoculating HEP53.4 cells mixed with macrophages (transfected with si-NC or si-NPLOC4, at a 2:1 ratio) and treated with IgG or CD8 antibody. n: number of biological replicates. *p < 0.05; **p < 0.01; ***p < 0.001.

To further explore the role of NPLOC4^+^ TAMs in HCC progression, we knocked down NPLOC4 in bone marrow-derived macrophages (BMDMs) and THP-1 cells using small interfering RNA (siRNA) (Figure S2A-B). Next, we established a subcutaneous tumor model by coinjecting HEP53.4 cells with NPLOC4-knockdown BMDMs. Additionally, through regular monitoring of tumor volume in the control groups (si-NC + HEP53.4) and experimental groups (si-NPLOC4 + HEP53.4), we observed significant delays in tumor growth and reduced tumor volumes in the NPLOC4-knockdown groups compared with the control groups (Figure 2A, Figure S2C). These findings suggest that NPLOC4^+^ TAMs exhibit a pronounced tumor-promoting effect. We subsequently investigated the interplay between NPLOC4^+^ TAMs and CD8^+^ T cells in murine tumors (Figure S2D). Following the knockdown of NPLOC4 expression in macrophages, we observed a significant increase in the number of CD8^+^ T cells infiltrating the tumor microenvironment (Figure 2B). Additionally, NPLOC4 knockdown led to a reduced exhaustion ratio (TIM-3^+^ PD-1^+^) of CD8^+^ T cells. (Figure 2C). Moreover, the tumor-killing activity of CD8^+^ T cells, as reflected by IFN-γ production, was inversely correlated with that of NPLOC4^+^ TAMs (Figure 2D). Furthermore, to further elucidate the impact of NPLOC4^+^ TAMs on the tumor microenvironment of HCC, we generated a macrophage-specific conditional knockout mouse model of Nploc4. Results from the orthotopic liver tumor-bearing model established with these mice demonstrated that tumor progression was significantly suppressed in the Nploc4^cKO^ group (Figure 2E). Immunofluorescence assays confirmed the complete ablation of Nploc4 expression in macrophages (Figure 2F). Flow cytometry showed that the deletion of NPLOC4 activated the CD8^+^ T cells in the TME (G-I). To further explore the role of CD8^+^ T cells in tumor progression mediated by NPLOC4^+^ TAMs, we employed a CD8 monoclonal antibody to deplete CD8^+^ T cells in mice (Figure S2D). By monitoring tumor volume and plotting growth curves, we observed that depletion of CD8^+^ T cells reversed the inhibitory effect of NPLOC4 knockdown in TAMs on tumor progression (Figure 2J-L). These findings indicate that NPLOC4^+^ TAMs drive hepatocellular carcinoma growth and progression by suppressing CD8^+^ T cell infiltration and function.

### NPLOC4 TAMs mediated protumor effects by promoting the transformation of M2 into M1 macrophages

**Figure 3:**
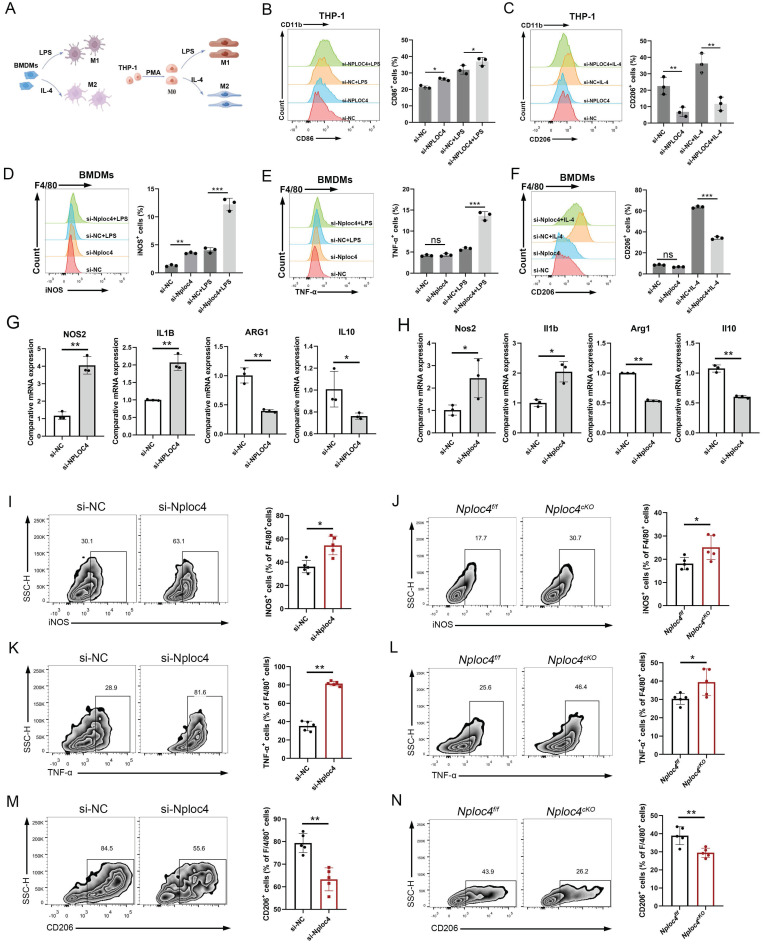
** NPLOC4 knockdown reprograms TAMs by promoting polarization of antitumoral macrophages.** (A) Schematic diagram of inducing macrophage M1/M2 polarization. (B-C) Representative histograms and percentages of CD86^+^ (B) and CD206^+^ (C) cells in THP-1 cells transfected with si-NC or si-NPLOC4. (D-F) Representative histograms and percentages of iNOS^+^ (D), TNF-α^+^ (E) and CD206^+^ (F) cells in BMDM cells transfected with si-NC or si-NPLOC4. (G-H) qPCR showing mRNA expression of M1 macrophage-related genes (NOS2 and IL-1β) or M2 macrophage-related genes (CD206 and IL-10) in BMDMs or THP-1 transfected with si-NC or si-NPLOC4. (I-J) Flow cytometry of the CD11b^+^F4/80^+^iNOS^+^ macrophages in NPLOC4 knockdown and control groups (n = 5 in each group). (K-L) Flow cytometry of the CD11b^+^F4/80^+^TNF-α^+^ macrophages in NPLOC4 knockdown and control groups (n = 5 in each group). (M-N) Flow cytometry of the CD11b^+^F4/80^+^CD206^+^ macrophages in NPLOC4 knockdown and control groups (n = 5 in each group). *P < 0.05, **P < 0.01, ***P < 0.001.

In tumor microenvironments, macrophages can be polarized to assume different phenotypes and play either pro- or antioncogenic roles 7. To gain a more detailed understanding of the functional mechanism of NPLOC4 in TAMs, we further conducted an in-depth analysis of the functional differences between NPLOC4^+^ TAMs and NPLOC4^-^ TAMs in the single-cell sequencing dataset GSE242889. The results of pathway enrichment analysis showed that the highly expressed genes in NPLOC4^+^ TAMs exhibited significant pathway enrichment in the PI3K-Akt signaling pathway, which is closely associated with M2 polarization of macrophages (Figure S3A). In sharp contrast, the highly expressed genes in NPLOC4- TAMs were enriched in the TNF-NFKB pathway, which is closely related to M1 polarization of macrophages (Figure S3B). Considering the relevance of the phenotypic polarization of macrophages in the tumor microenvironment, we next knocked down the expression of NPLOC4 in BMDMs and THP-1 cells through si-NPLOC4, and used LPS and IL-4 to mediate M1/M2 polarization respectively (Figure 3A). The results of flow cytometry revealed that knockdown of NPLOC4 promoted M1 polarization (increased expression of iNOS, TNF-α, and CD86) and inhibited M2 polarization (decreased expression of CD206) in both BMDMs and THP-1 cells (Figure 3B-F, Figure S4A). Quantitative PCR (qPCR) analysis confirmed that the mRNA expression levels of M1 polarization markers, including Nos2 and Il-1β, were significantly upregulated, whereas the expression levels of M2 polarization markers, including Arg1 and Il10, were significantly decreased (Figure 3G-H). Consistently, flow cytometry revealed more M1 macrophages (expressing iNOS and TNF-α) and fewer M2 macrophages (expressing CD206) in the tumor tissues from the NPLOC4 knockdown group (Figure 3I-N). Furthermore, we co-cultured NPLOC4-knockdown BMDMs with Hepa1-6 or HEP53.4 cells, the results indicated that NPLOC4 knockdown strongly slowed tumor cell proliferation (Figure S4B). Thus, these results indicated that knock down of NPLOC4 in macrophages facilitates TAM polarization into the M1 subtype in HCC.

### NPLOC4 regulated the type I interferon pathway in macrophages

**Figure 4:**
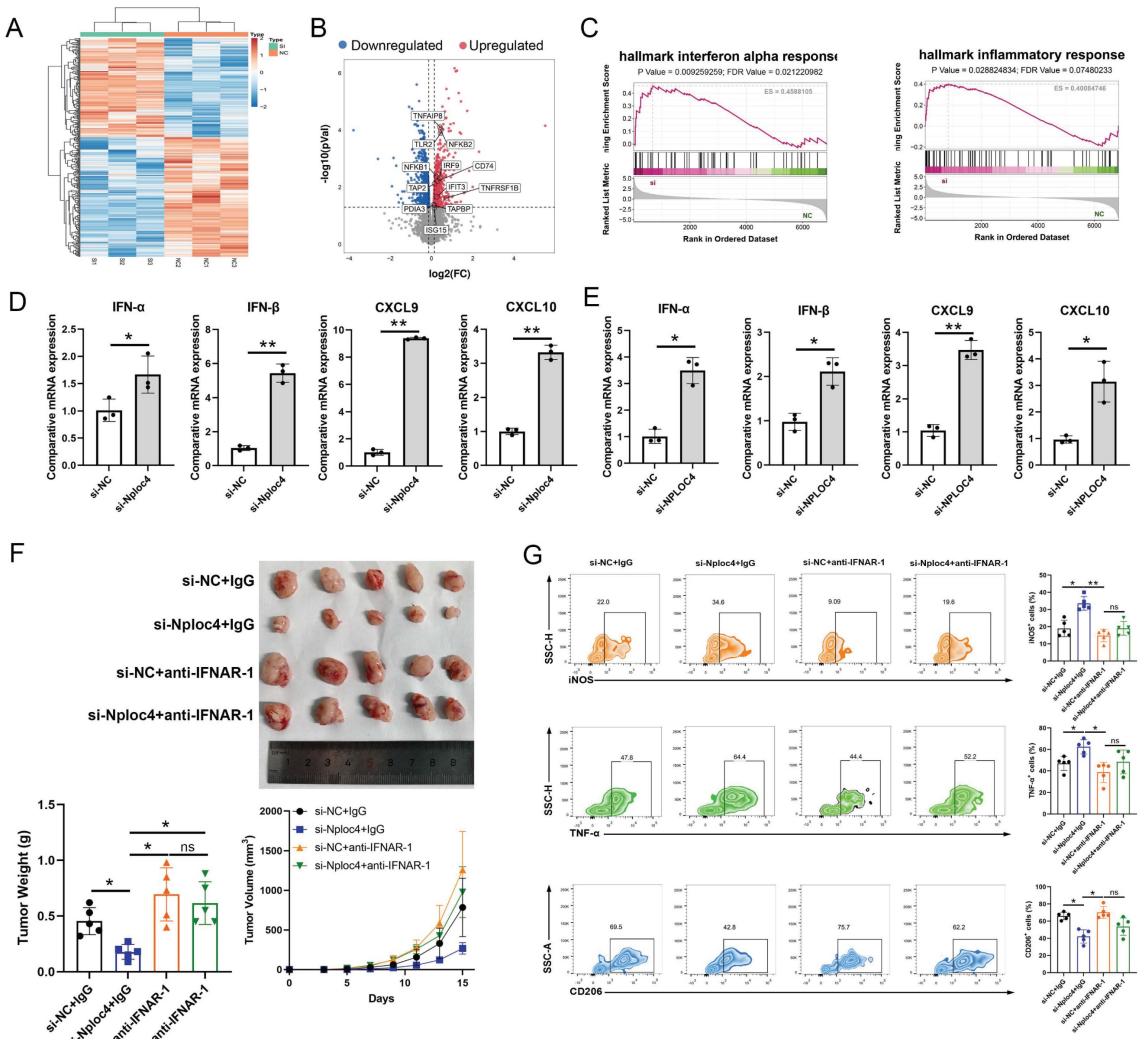
** NPLOC4^+^ TAMs mediate the pro-tumor effects by inhibiting type I interferon pathway.** (A) Heatmap of different expressed proteins of THP-1 cells transfected with si-NC or si-NPLOC4. (B) Volcano map showed the type I inteferon related genes were upregulated in NPLOC4 knockdown THP-1 cells. (C) GSEA results of the IFN-α response in NPLOC4 knockdown THP-1 cells compared to negative controls. (D-E) qPCR showing mRNA expression of type I interferon related genes in BMDMs (D) or THP-1 cells (E) transfected with si-NC or si-NPLOC4. (F) The diagram, tumor volume and weight of tumor growth in mouse model inoculating HEP53.4 cells mixed with macrophages (transfected with si-NC or si-NPLOC4, at a 2:1 ratio) and treated with IgG or IFNAR1 antibody (n=5). (G) Flow cytometry of the macrophages expressing iNOS, TNF-α or CD206 in the mouse model. n: number of biological replicates. *p < 0.05; **p < 0.01; ***p < 0.001.

To further explore the potential mechanisms through which NPLOC4 regulates macrophage polarization, we further performed proteomic sequencing data on BMDMs transfected with si-NC and si-NPLOC4. Proteomic analysis of the two cell groups identified a substantial number of differentially expressed proteins (Figure 4A). Volcano map analyses revealed the upregulation of type I interferon and inflammatory response associated genes (Figure 4B). GSEA analysis revealed that the type I interferon and antigen presentation pathway (Figure 4C). The type I interferon pathway is a vital regulatory pathway for M1 type polarization of macrophages 24. Thus, we hypothesized that NPLOC4^+^ TAMs promote tumor progression by suppressing type I interferon secretion. Hence, we detected the expression levels of type I interferon associated genes, including IFN-α, IFN-β, CXCL9, CXCL10 using qPCR. In line with the proteomic sequencing findings, the expression levels of type I interferon and a panel of downstream cytokines in macrophages were notably elevated in macrophages with NPLOC4 knockdown (Figure 4D-E). To validate the critical role of type I interferon in NPLOC4^+^ TAM-mediated immunosuppression, we utilized a specific antibody to block the type I interferon receptor IFNAR1. Analysis of tumor growth showed that anti-IFNAR1 treatment significantly reduced tumor growth suppression compared to the control group (Figure. 4F). Concurrently, flow cytometry analysis indicated that blockade of IFNAR1 partially reversed the NPLOC4 knockdown-induced increase in M1 macrophage infiltration and functional activation (Figure 4G). Therefore, NPLOC4 may regulate macrophage polarization by inhibiting the type I interferon pathway.

### NPLOC4 mediates ubiquitin-dependent degradation of RIG-1 to inhibit the type I interferon pathway

**Figure 5:**
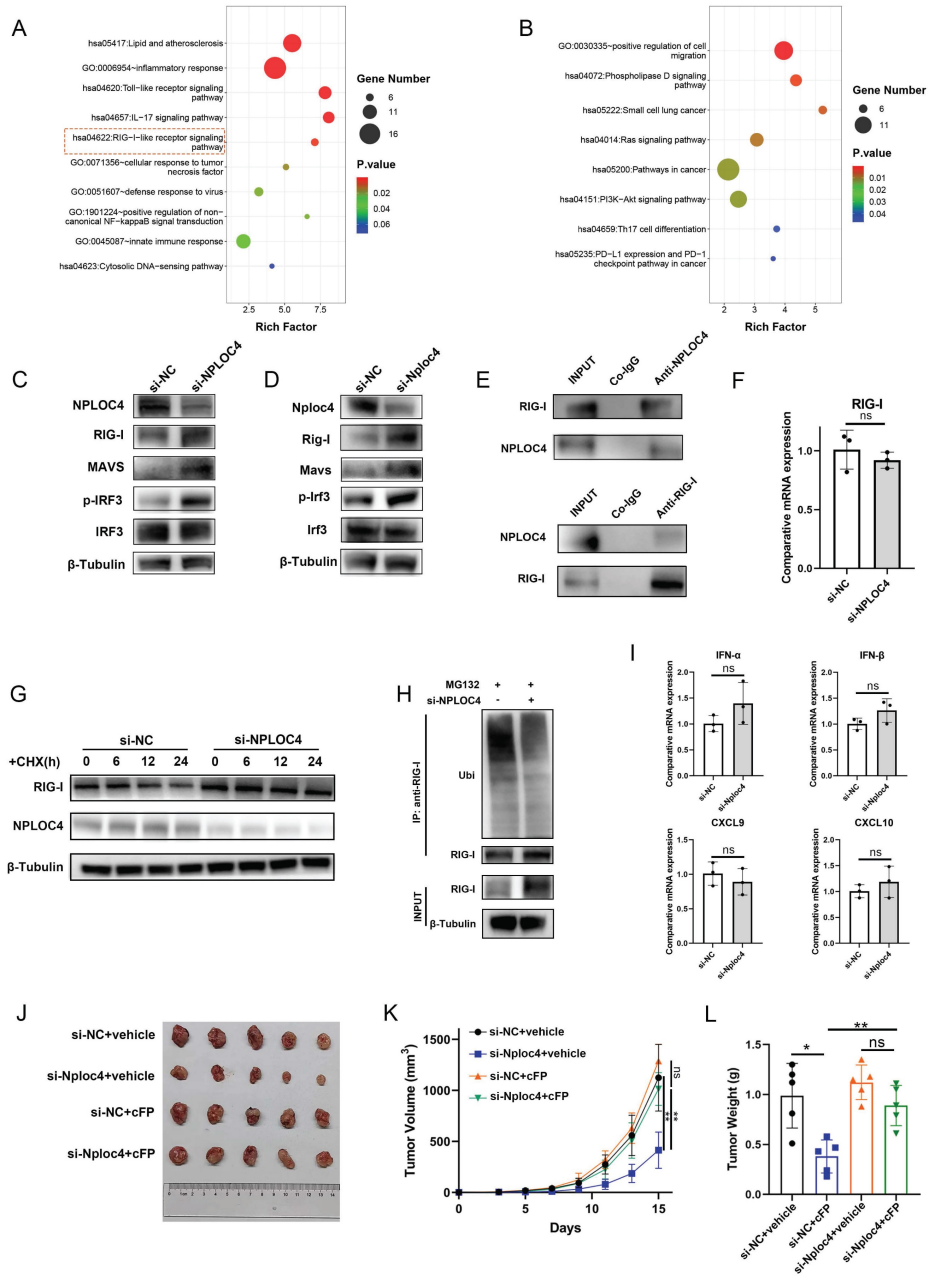
** NPLOC4 mediate IFN-α inhibition by facilitating proteasomal degradation of RIG-I.** (A-B) Function enrichment of up-regulated (A) and down-regulated (B) proteins of NPLOC4 knockdown macrophages. (C-D) Immunoblot analysis of RIG-I pathway of BMDMs and THP-1 cells transfected with si-NC or si-NPLOC4. (E) Immunoprecipitation assay for the interaction between NPLOC4 and RIG-I proteins in THP-1 cells. (F) qPCR showing mRNA expression of RIG-I in THP-1 transfected with si-NC or si-NPLOC4. (G) Protein levels of RIG-I in THP-1 cells knocking down NPLOC4 after being treated with cycloheximide (CHX) (200 μg/mL). (H) Ubiquitination of RIG-I in THP-1 cells transfected with si-NC or si-NPLOC4. (I) qPCR showing mRNA expression of type I interferon related genes in THP-1 cells treated with RIG-I inhibitor Cyclo(Phe-Pro) (cFP). (J-L) The diagram (J), tumor volume (K) and weight (L) of tumor growth in mouse model inoculating HEP53.4 cells mixed with macrophages (transfected with si-NC or si-NPLOC4, at a 2:1 ratio) and treated with vehicle or cFP (50 mg/kg) (n=5). n: number of biological replicates. *p < 0.05; **p < 0.01; ***p < 0.001.

Collectively, the results from previous experiments demonstrated that NPLOC4 inhibited the type I interferon signaling pathway in TAMs; therefore, we explored how type I interferon signaling is inhibited. We further analyzed the proteomics data. The results of pathway enrichment showed that the genes up-regulated after NPLOC4 knockdown were enriched in the RIG-I pathway (Figure 5A-B). The RIG-I/MAVS pathway represents a canonical intracellular RNA-sensing pathway that triggers type I interferon secretion 25. Previous studies have indicated that NPLOC4 can regulate the type I interferon pathway through the RIG-I signaling 26. Thus, we evaluated activation of the RIG-I/MAVS pathway in NPLOC4-knockdown TAMs by western blot. Following NPLOC4 knockdown, intracellular RIG-I protein levels were significantly elevated, along with MAVS proteins and phosphorylation of downstream IRF3 proteins (Figure 5C-D). Thus, we hypothesized that NPLOC4^+^ TAMs might suppress RIG-I/MAVS pathway activation, thereby mediating reduced type I interferon secretion. Additionally, we detected an interaction between endogenous NPLOC4 and endogenous RIG-I in THP-1 cells (Figure 5E). Considering that NPLOC4 and RIG-I correlate negatively, there might be a regulatory mechanism between them. Nevertheless, qRT-PCR analysis revealed that NPLOC4 knockdown did not significantly alter RIG-I mRNA levels (Figure 5F). These findings suggest that NPLOC4 modulates RIG-I predominantly through post-translational mechanisms rather than transcriptional regulation. To validate NPLOC4-mediated regulation of RIG-I protein levels, we conducted a half-life assay. The results showed that RIG-I exhibited a reduced half-life and accelerated degradation in THP-1 cells transfected with si-NC compared to NPLOC4-knockdown cells (Figure 5G). Subsequently, we assessed the effect of NPLOC4 knockdown on RIG-I ubiquitination. The results indicated that RIG-I ubiquitination decreased markedly in the NPLOC4 knockdown cells compared with that in the control cells (Figure 5H). Furthermore, we used the RIG-I inhibitor Cyclo(Phe-Pro) (cFP) to suppress the RIG-I pathway in macrophages. The results showed that the use of cFP eliminated the activation of the type I interferon mediated by NPLOC4 knockdown (Figure 5I). Meanwhile, inhibition of RIG-I function in NPLOC4-knockdown macrophages can eliminate the promoting effect of NPLOC4 knockdown on M1-type polarization of macrophages (Figure S5A-B). Moreover, activating the RIG-I pathway via 3pRNA can mimic the regulatory effect of NPLOC4 knockdown on macrophage polarization, suggesting that the activation of RIG-I is a core link in the induction of M1-type polarization by NPLOC4 knockdown (Figure S5C-D). Ultimately, *in vivo* mouse experiments validated the role of RIG-I in tumor immunosuppression mediated by NPLOC4^+^ macrophages. Our findings showed that RIG-I blockade significantly reversed the tumor growth inhibitory effect of NPLOC4 knockdown (Figure 5J-L).

To explore the underlying mechanism of NPLOC4-related degradation of RIG-I, we set out to identify the potential E3 ligase responsible for RIG-I turnover during this process. So far, RNF125 has been identified to mediate NPLOC4-related K48-linked ubiquitination of RIG-I. We knocked down the expression level of RNF125 in THP-1 cells using siRNA. The results of PCR showed that RNF125 knockdown could mediate the expression of genes of the type I interferon pathway (Figure S6A). Western blot indicated that RNF125 knockdown could increase the protein level of RIG-I (Figure S6B). Additionally, we detected an interaction between endogenous RNF125 and endogenous RIG-I in THP-1 cells (Figure S6C). We next examined the ubiquitination levels of RIG-I in cells transfected with si-NPLOC4 and/or RNF125. Consistent with the aforementioned results, inhibition of NPLOC4 significantly decreased K48-linked ubiquitination of RIG-I, while co-inhibition of NPLOC4 with RNF125 further enhanced this effect, indicating that NPLOC4 acts synergistically with RNF125 to facilitate K48-linked ubiquitination of RIG-I (Figure S6D). Taken together, our findings indicated that NPLOC4 promotes RIG-I degradation by promoting RNF125-mediated K48-linked ubiquitination of RIG-I.

### Inhibiting NPLOC4 significantly augmented the anti-tumor immune response and potentiated the sensitivity of liver cancer to PD-1 therapy

**Figure 6:**
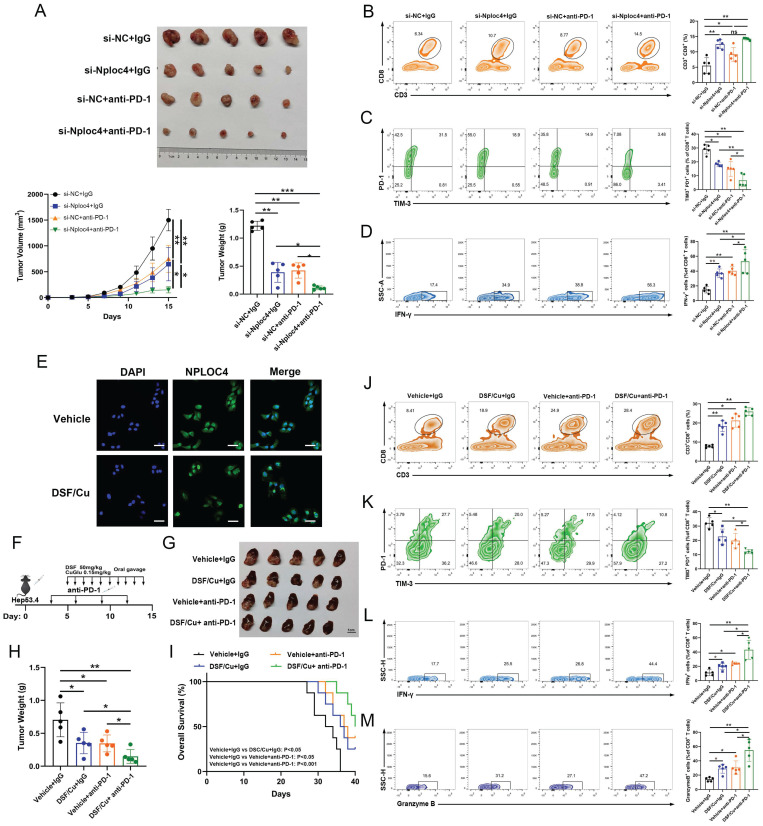
** NPLOC4 inhibition significantly improves the anti-tumor immune response and enhances liver cancer response to PD-1 therapy.** (A) The diagram, tumor volume and weight of tumor growth in mouse model inoculating HEP53.4 cells mixed with macrophages (transfected with si-NC or si-NPLOC4, at a 2:1 ratio) and treated with IgG or PD-1 antibody (n=5). (B) Flow cytometry of CD8^+^ T cell ratio to CD45^+^ in four groups of tumor model. (C) Flow cytometry of the exhausted status of CD8^+^ T cells (PD-1^+^ and TIM-3^+^ labeling) in four groups of tumor model. (D) Flow cytometry of the cytotoxic function of CD8^+^ T cells (IFN-γ labeling) in four groups of tumor model. (E) Fluorescence imaging of NPLOC4 in THP-1 cells treated with disulfiram/copper (DSF/Cu). Scale bar: 50 μm. (F) Protocol schematic of the combination of DSF/Cu and anti PD-1 therapy in an orthotopic tumor model. (G-H) The diagram (G) and tumor weight (H) of tumor growth of four groups of orthotopic tumors (n=5). (I) Survival curve analysis of four groups of mice (n=8). (J) Flow cytometry of CD8^+^ T cell ratio to CD45^+^ in four groups of tumor model. (K) Flow cytometry of the exhausted status of CD8^+^ T cells (PD-1^+^ and TIM-3^+^ labeling) in four groups of tumor model. (L-M) Flow cytometry of the IFN-γ^+^ (L) and Granzyme B^+^ (M) cells in four groups of tumor model. n: number of biological replicates. *p < 0.05; **p < 0.01; ***p < 0.001.

**Figure 7:**
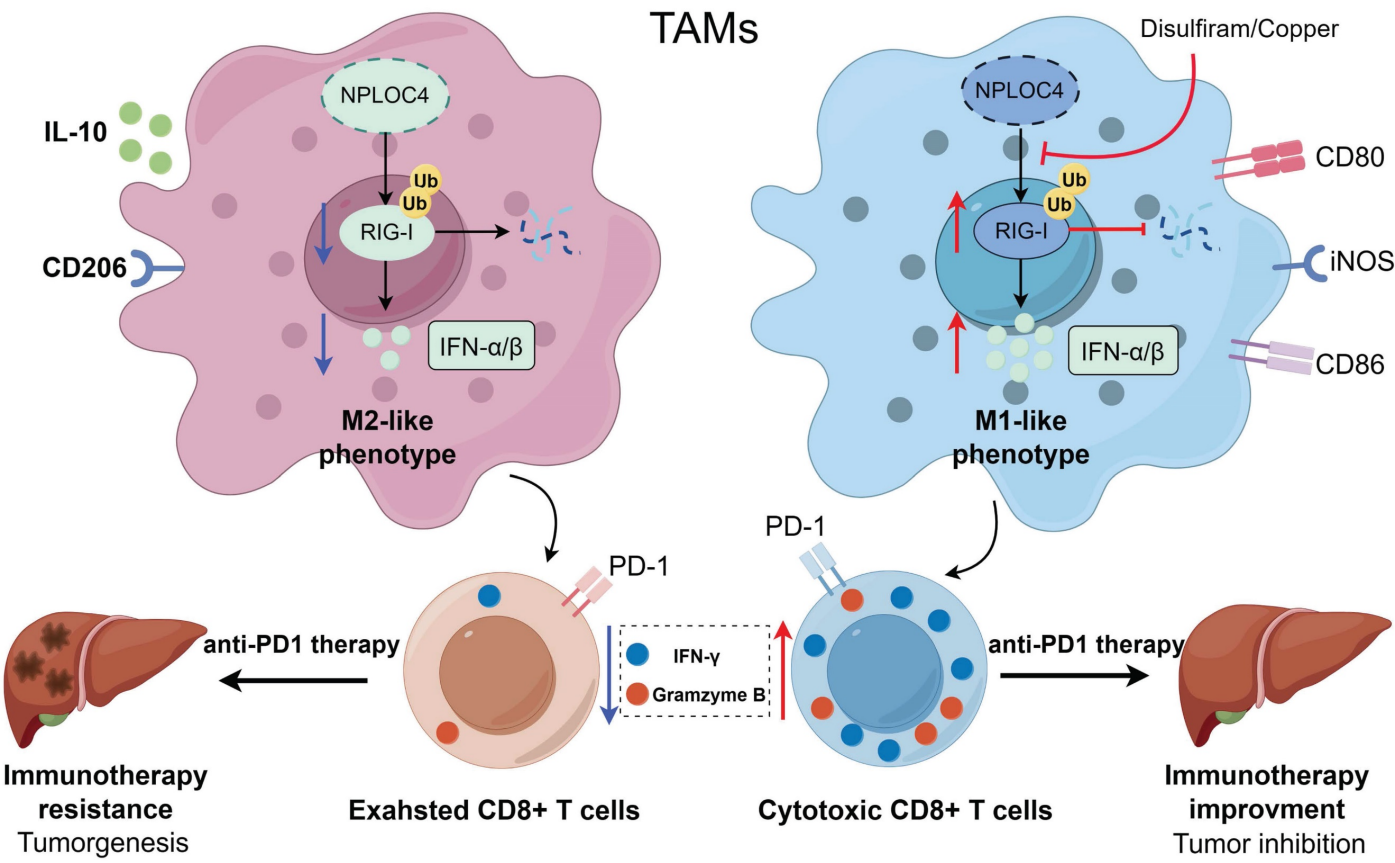
** A working model for how NPLOC4 reduction reprograms TAMs and elicits potent anti-tumor immunity.** NPLOC4 helps to promote ubiquitination and proteasomal degradation of RIG-I, thereby driving the polarization of macrophages toward the M2 phenotype and facilitating immune escape in HCC. Knockdown of NPLOC4 enhances the protein level of RIG-I, reprograms TAMs to induce anti-tumoral polarization, thereby facilitating CD8^+^ T cell anti-tumor response, and eventually suppressing tumor growth.

The infiltration and functionality of CD8^+^ T cells within tumors play a pivotal role in determining the effectiveness of anti-PD-1 and other immune checkpoint blockade therapies 27-28. Our prior work demonstrated that silencing NPLOC4 in TAMs led to a notable increase in both the quantity and functionality of infiltrating CD8^+^ T cells in HCC. Subsequently, we co-administered si-NPLOC4-transfected BMDMs with a PD-1 antibody to assess whether NPLOC4 inhibition in macrophage subsets could potentiate the efficacy of anti-PD-1 therapy. The most potent tumor suppressive effect was observed when si-NPLOC4 was combined with anti-PD-1 therapy (Figure 6A). Flow cytometry showed that the inhibition of NPLOC4 increased the population of CD8^+^ T cells in the TME (Figure 6B). Importantly, the combined therapy decreased the expression of the co-inhibitory molecules PD-1 and TIM3 in CD8^+^ T cells isolated from the tumors (Figure 6C). Meanwhile, the IFN-γ secretion by CD8^+^ T cells also increased (Figure 6D). Furthermore, studies have indicated that disulfiram/copper (DSF/Cu) could target the NPLOC4 protein 29-30. We further treated BMDMs with DSF/Cu. In line with prior investigations, our results from immunofluorescence analyses also revealed nuclear aggregation and accumulation of NPLOC4 following DSF/Cu treatment (Figure 6E). Furthermore, to verify whether DSF/Cu treatment could enhance the immune response of hepatocellular carcinoma, we constructed orthotopic parental and HEP53.4 cell-bearing mice combined with anti-PD-1 therapy (Figure 6F). *In vivo* results from mouse orthotopic tumor models showed that DSF/Cu and anti-PD-1 therapies exerted significant synergistic effects, leading to marked inhibition of HCC progression (Figure 6G-H). Additionally, survival analysis of tumor-bearing mice demonstrated that the combination therapy notably enhanced the prognosis of the mice (Figure 6I). The activation of CD8^+^ T cells was also observed in the combination therapy group (Figure 6J-M). Thus, targeting NPLOC4^+^ macrophages can markedly enhance the efficacy of anti-PD-1 therapy in liver cancer, holding promise as a novel potential target for combination immune checkpoint inhibitor therapy (Figure 7).

## Discussion

The primary cytotoxic response against malignant cells is mediated by CD8^+^ T lymphocytes that penetrate the tumor niche. Nevertheless, cancer cells establish an immunoinhibitory milieu through multiple pathways to escape elimination by these cytotoxic T cells. Current immunotherapeutic strategies predominantly center on regulating CD8^+^ T-cell reactivity and often neglect the pivotal contributions of innate immune elements within the tumor microenvironment. Numerous studies have shown that most TAMs within the tumor microenvironment possess robust immunosuppressive capabilities. Nonetheless, owing to their heterogeneity, these cells necessitate more precise exploration in future studies. Our findings indicate that a subset of macrophages with elevated NPLOC4 expression levels can facilitate HCC development and progression by decreasing CD8^+^ T cell infiltration and suppressing their tumoricidal activity within the HCC microenvironment. Prior investigations have demonstrated that NPLOC4 modulates the tumor immune microenvironment by promoting the differentiation of immunosuppressive Tregs and mediating glioblastoma cell resistance to cytotoxic T cells 16, 31. Nonetheless, limited research has explored how NPLOC4^+^ TAMs influence the infiltration and functionality of these cells, and the underlying mechanism remains undefined.

To the best of our knowledge, this study represents the first report linking the oncogenic functions of NPLOC4^+^ TAMs to type I interferon signaling pathways. Specifically, macrophages with elevated NPLOC4 expression exhibit reduced type I interferon production, promoting the M2 polarization of macrophages and inhibiting specific immune activation. Initially identified for its ability to combat viral infections, type I interferon has subsequently emerged as a significant immunoregulatory molecule with expanding recognized functions in non-viral infectious diseases 32. Type I interferons exhibit direct antitumor activity by inhibiting tumor growth, inducing apoptosis, and mediating indirect cytotoxic effects through immune cell activation 33-34. Nevertheless, the limited presence of type I interferons within tumors suggests their primary mechanism may involve autocrine-mediated induction of M1-polarized macrophages. Additionally, as a downstream effect of type I interferon, TAMs with high NPLOC4 expression exhibit characteristics of an activated PI3K-AKT pathway and high expression of M2 polarization markers (CD206), whereas the NF-κB pathway suppressed and M1 polarization markers (iNOS and TNF-α) decreased, leading to decreased recruitment, infiltration, and restricted CD8^+^ T cell activation, findings that align with our results.

As one of the most multifunctional cofactors of p97, NPLOC4 is involved in more than half of the cellular processes mediated by p97, including endoplasmic reticulum-associated degradation (ERAD) 35. During the ERAD process, p97 recruits NPLOC4 to extract polyubiquitinated proteins from the ER membrane. This is followed by processing the extracted proteins and delivering them to the proteasome for degradation 36. Previous research has demonstrated that NPLOC4 suppresses type I interferon production by enhancing ubiquitin-dependent proteolysis of RIG-I, consequently attenuating host antiviral defenses 26. Because RIG-I triggers the release of IFN-I, activation of the RIG-I pathway significantly affected on the functions of various innate immune cells. With respect to dendritic cells (DCs), activation of the RIG-I pathway induces DC maturation, upregulates the expression of major histocompatibility complex (MHC) molecules and costimulatory molecules (such as CD80 and CD86), enhances the ability of DCs to take up, process, and present tumor antigens, and promotes the DC-mediated activation of naive T cells to initiate adaptive immune responses 37. In a mouse model of acute kidney injury, activation of the RIG-I pathway promotes macrophage polarization to the M1 phenotype via the pyroptosis pathway 38. In this study, we demonstrated that in macrophages, NPLOC4 helps to promote ubiquitination and proteasomal degradation of RIG-I, thereby driving the polarization of macrophages toward the M2 phenotype and facilitating immune escape in HCC. As for the specific mechanism by which NPLOC4 mediates RIG-I ubiquitination, Qian et al. have confirmed that the NPLOC4-p97 complex can directly interact with RIG-I and the E3 ubiquitin ligase RNF125 26. It specifically promotes K48-linked ubiquitination of RIG-I, thereby facilitating its entry into the proteasomal degradation pathway. Consistent results have also been obtained in the present study.

Numerous studies have demonstrated that the presence and activity of CD8^+^ T cells within the tumor microenvironment are frequently critical for the efficacy of immune checkpoint blockade therapy 39. Our results showed that NPLOC4^+^ macrophages in HCC suppressed CD8^+^ T cell infiltration and functionality by inhibiting the type I interferon response. This mechanism may underlie the poor response of HCC to monotherapy with anti-PD-1 agents. Disulfiram, initially emerging as an anti-alcoholism drug, when combined with copper ions, the resulting complex can specifically act on NPLOC4, exerting anti-cancer effects by interfering with its normal biological functions 30. Indeed, research has shown that disulfiram can create a favorable milieu for tumor immunotherapy by modulating the tumor immune microenvironment. Yuya and colleagues reported that disulfiram decreases macrophage tumor-promoting activity by blocking the interaction between FROUNT and CCR2/CCR5 [40. Another study indicated disulfiram significantly potentiates dendritic cells (DCs) maturation through activation of cGAS/STING pathway in a mouse model 41. Furthermore, ongoing clinical trials are assessing the efficacy and safety of DSF alone and in combination with Cu across various cancer types 42-43. It has also been shown in studies that DSF/Cu can be combined to enhance the efficacy of current first-line treatment regimens, such as sorafenib and lenvatinib for HCC 44-45. Consequently, we inhibited NPLOC4 using DSF/Cu and observed a notable increase in CD8^+^ T cell infiltration within the tumor microenvironment, accompanied by enhanced CD8^+^ T cell functionality. The combination of DSF/Cu and anti-PD-1 antibodies exerted a significant synergistic effect, markedly decelerating liver cancer progression. Collectively, our findings offer a novel combinatorial strategy for immune checkpoint therapy, aiming to enhance sensitivity and response rates in patients with liver cancer.

This study has limitations in that it mainly relies on Hepa1-6 and HEP53.4 xenograft models, and does not use spontaneous liver cancer models or liver cancer models with stronger immunogenicity (such as diethylnitrosamine (DEN)-induced models).

## Conclusion

In summary, our research validated that the immunosuppressive effect of NPLOC4^+^ TAMs arises from the inhibition of the type I interferon pathway. The study further elucidated the specific mechanism and confirmed the essential role of RIG-I therein. Additionally, suppressing the NPLOC4^+^ TAM subpopulation can effectively ameliorate the immunosuppressive tumor microenvironment and enhance the efficacy of immune checkpoint therapy, thus offering a novel potential target for liver cancer immunotherapy.

## Supplementary Material

Supplementary methods, figures and tables.

## Data Availability

The data will be made available on reasonable request. The mass spectrometry proteomics data have been deposited to the ProteomeXchange Consortium via the iProX partner repository with the dataset identifier PXD065671.

## Abbreviations

BMDMs: bone marrow-derived macrophages
cFP: Cyclo(Phe-Pro)
DCs: dendritic cells
DEN: diethylnitrosamine
DSF/Cu: disulfiram/copper
ERAD: endoplasmic reticulum associated degradation
HCC: hepatocellular carcinoma
ICB: immune checkpoint blockade
MHC: major histocompatibility complex
NPLOC4: NPL4 homolog, ubiquitin recognition factor
PD-1: programmed cell death protein-1
TACE: transarterial chemoembolization
TAMs: tumor-associated macrophages
TME: tumor microenvironment
Tregs: regulatory T cells
