# Supplementary material for: NPLOC4 Inhibition Remodels Tumor Microenvironment via M2-to-M1 Macrophage Reprogramming and Boosts Anti-PD-1 Response in Liver Cancer
Source: Int J Biol Sci. 2026 Feb 11;22(5):2687–701. doi: 10.7150/ijbs.125201 (PMC12965249; doi:10.7150/ijbs.125201)
Supplement: Supplementary file 1 — Supplementary methods, figures and tables. [file ijbsv22p2687s1.pdf]

Supplementally Methods

Mice

C57BL/6J mice were purchased from Ziyuan Biotechnology Co, Ltd (Hangzhou, China). Animal experiments were approved by the Ethics Committee of First Affiliated Hospital of Zhejiang University, School of Medicine (2024-69).

Cell culture and transfection

Hepa1-6 and THP-1 was obtained from America Type Culture Collection (Manassas, VA) in 2017. HEP-53.4 was obtained from Cell Line Service GmbH in 2023. Cells were cultured in Dulbecco's Modified Eagle Medium (DMEM, Life Technologies, Gaithersburg, MD supplemented with 10% FBS, 1% penicillin/streptomycin solution).

The Cell line authentication was conducted at the same time. Cell-line authentication was performed simultaneously. In addition, BMDMs extracted from the thigbones of C57BL/6 mice were cultured in the medium mentioned above for 7 d alongside M-CSF (25 ng/ml, PeproTech) for further experiments.

For knockdown assays, the NPLOC4 siRNA and negative control siRNA (Ruibo, Hangzhou, China) were transfected into the BMDMs at 110 nmol/L using jetPRIME® Transfection Reagent (101000046, Polyplus) at 4 μL.

Analysis of public transcriptome datasets

Three public transcriptome datasets were enrolled in our study, including the TCGA-LIHC cohort of the Cancer Genome Atlas (n=365) and the CHCC-HBV cohort of Gao et al (n=159). Among above datasets, any case with null value of survival information had been excluded. The fragments per kilobase per million (FPKM) data and clinic information were downloaded from the UCSC Xena (xenabrowser.net) and The National Omics Data Encycolpedia (www.biosino.org/node), respectively. All FPKM values were transformed into transcripts per kilobase million (TPM) values. Utilizing the TIMER 2.0 website, we estimated the macrophage infiltration level of each patients in the two datasets.

Analysis of public sc-RNA datasets

The human HCC scRNA-seq datasets were downloaded from GEO database (GSE202642 and GSE242889) Cells with less than 500 or more than 20,000 UMIs, over 20% mitochondrial genes and more than 0.1% hemoglobin genes were filtered out. "Seurat" (Version 4.3.0) was applied for data normalization, finding variable genes, scaling (NormalizeData, FindVariableFeatures and ScaleData functions), principal component analysis (RunPCA), dimension reduction (RuntSNE) and unsupervised graph-based clustering (FindNeighbors & FindClusters). "Harmony" (Version 0.1) was used to remove batch effect (RunHarmony) . Differentially expressed genes (DEGs) for each cluster were identified (FindAllMarkers) andvisualized via tSNE plots, heatmaps, violin plots and volcano plots. "scRNAtoolVis" (Version 0.0.4) was used to plot markers in each cluster (jjVolcano) with an absolute cutoff value of 0.5 for log2 fold change and a threshold of 0.01 for adjusted p-value. DEGs between the specified clusters were identified (FindMarkers).

Cell proliferation assays

Cell proliferation capacity was measured by Cell Counting Kit (CCK8, MCE) and colony formation assay. In terms of CCK8, 2.0×10^{3} cells were seeded into 96-well plates with five duplicates. CCK8 reagents were diluted with DMEM at a ratio of 1:9 and 100 μL for each well. The 450 nm absorbance was detected after 1 h incubation at 37 °C in each day for consecutive five days, followed by the establishment of cell viability curves.

Establishment of mouse tumor model and treatment

To establish the orthotopic model, 25uL mixture of RPMI1640 and Basement Membrane Matrix (with the ratio of 1:1, 354248, Corning, Matrigel) containing 0.5×10^{6} Hepa1-6 cells/HEP 53.4 cells were injected into the left liver lobe of male C57BL/6J mouse (6-8 weeks old). To establish a subcutaneous tumor model, 50 uL RPMI1640 containing 0.5×10^{6} Hepa1-6 cells/HEP 53.4 cells and 0.25×10^{6} BMDM cells were subcutaneously injected into the mouse on the left flank. Subcutaneous

tumor volumes were calculated using the modified ellipsoidal formula: V= 1/2 (length×width^2).

For the CD8 blockade experiment, mice were treated with anti-mouse CD8α (12.5 mg/kg, BE0061, BioXcell) via intraperitoneal injection every 3d following inoculation for 3d.

For the IFNAR1 blockade experiment, mice were treated with anti-mouse IFNAR-1 (25 mg/kg, BE00241, BioXcell) via intratumor injection every 3d after inoculation for 3d.For the RIG-I inhibition experiment, mice were received oral administration of Cyclo(Phe-Pro) (cFP, 50mg/kg, HY-P1934, MedChemExpress) every 3d after inoculation for 3d.

Mice were intraperitoneally injected with anti-PD-1 (20 mg/kg, BE0361, BioXcell) every 3d after inoculation for 3d.

For the DSF/Cu treatment, mice were received oral administration of 0.15mg/kg CuGlu (Sangon Biotech, A503014) and 50mg/kg DSF (Selleck, S1680) every day. CuGlu was administered 2 hours before DSF.

Flow Cytometry Staining

Cells were stained with Zombie NIR™ Fixable Viability Kit（423106), CD45 PE (147711), CD45 FITC (147709), CD3 APC (100235), CD8 FITC (100705), IFN-γ Percp-cy5.5 (505821), PD-1 PE-Cy7 (135215), TIM-3 Percp-cy5.5 (134011), Granzyme B PE-Cy7 (396410), CD11b Percp-cy5.5(101228), F4/80 FITC (123108), CD86 PE-Cy7(105014), iNOS PE (696805), TNF-α, CD206 PE(321105), CD206 APC (321109), CD86 PE (374205), CD11b FITC (982614) ,Unless otherwise specified, all the reagents were from Biolegend.

Immunohistochemistry and immunofluorescence

For immunofluorescence, tissue sections were subjected to antigen retrieval using an induction cooker at pH 9.0 in EDTA buffer for 25 minutes. The following antibodies were incubated with tissue sections after treatment with goat serum at 37°C for 40

minutes: NPLOC4 (ab239369, Abcam), CD68 (ab955, Abcam), F4/80 (NB600-404, Novus) at 4 °C overnight. The slides were incubated with Alexa Fluor 488,594 secondary antibodies (A11008, A11001). Immunostaining was visualized under an immunofluorescence microscope and it was evaluated using the Image-Pro Plus software (Media Cybernetics, Silver Springs, MD, USA). For immunoprecipitation(IP), IP/Western lysing solution (P0013, Beyotime Biotechnology) was used to lyse the cells and then IP was performed according to the manufacturer's instructions followed western blotting detection experiments. The quantitative results of western blotting were obtained using ImageJ1.8.0 software (NIH, Bethesda, MD, USA).

Western blotting analysis and immunoprecipitation

For western blotting, protein was extracted (Minute SN-001, Invent Biotechnologies, Inc, Beijing, China) and via 8–15% SDS-PAGE electrophoresis, after transferred to PVDF membranes it was incubated with antibodies against NPLOC4 (11638-1-AP, Proteintech), NPLOC4 (sc-365796, santa cruz), RIG-I (Proteintech, 25068-1-AP),TBK1 (#3504, CST),IRF3 (#4302, CST), phosphor-TBK1 (#5483, CST), phosphor-IRF3 (#29047, CST), Ubiquitin (A0162, abclonal), MAVS (A5764, abclonal), RNF125 (13290-1-AP, Proteintech)β-Tubulin (A12289, abclonal) and β-Actin (A00702, Genscript). All primary antibodies were purchased from Cell Signaling Technology (Danvers, MA, USA). As a secondary antibody, HRP-conjugated anti-rabbit or anti-mouse antibodies were used, and the enhanced chemiluminescence assay (ECL, Thermo, Waltham, MA) was used to visualize the antigen-antibody reaction.

Targeted proteomic

Samples were taken from the -80°C refrigerator and inserted on ice to thaw, then pre-mixed lysis solution (8 M urea, 1% protease inhibitor) was slowly added to the sample tubes and lysed by sonication in an ultrasonic crusher. Centrifugation (4°C, 15000 g, 10 min) was performed to remove cellular debris, and the supernatant was

transferred to a new centrifuge tube, and the supernatant protein concentration was determined using the BCA method. After the procedures of Precipitation enzyme digestion, FASP digestion, Tandem Mass Tag (TMT) labeling, and HPLC Fractionation, the sample was loaded onto the sequencing instrument for sequencing. The analysis utilizes the nanoflow rate Vanquish Neo system (Thermo Fisher Scientific) for chromatographic separation, and samples post high-efficiency liquidchromatography separation are subjected to Data Independent Acquisition (DIA) mass spectrometry using the Astral high-resolution mass spectrometer (Thermo Scientific). The detection mode is positive ion, with a precursor ion scan range of 380-980 m/z, and a primary mass spectrometry resolution of 240,000 at 200 m/z. Normalized AGC Target is set at 500%, with a Maximum IT of 5ms. MS2 employs the DIA data acquisition mode, with 299 scan windows set, an Isolation Window of 2m/z, HCD Collision Energy set at 25ev, Normalized AGC Target at 500%, and a Maximum IT of 3ms. The DIA data is processed using the DIA-NN software. The software parameters are set as follows: the enzyme used is trypsin, with a maximum missed cleavage site set to 1. Fixed modification is Carbamidomethyl (C), and dynamic modifications are set to Oxidation (M) and Acetyl (Protein N-term). Proteins identified through database retrieval must pass the set filtering parameter of FDR<1%.

Supplementary Figures

Figure S1

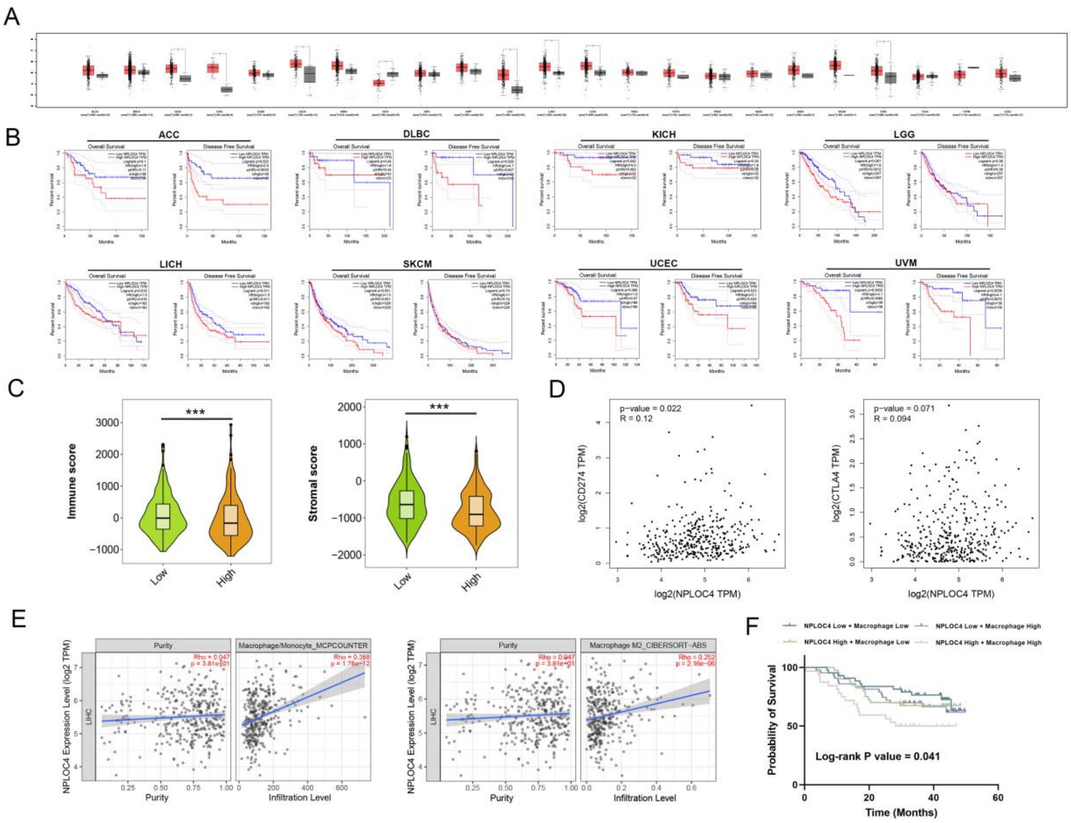

Figure S1. The expression pattern of NPLOC4 in cancers and tumorinfiltrating macrophages. (A) Boxplots showing the NPLOC4 expression in multiple types of cancers and corresponding normal tissues from RNA-seq results of the TCGA database. (B) Prognosis analysis of NPLOC4 in multiple types of cancers from RNA-seq results of the TCGA database. (C) Boxplots showing the immune and stromal scores calculated by ESTIMATE algorithms between low and high NPLOC4 expression groups from the TCGA database. (D) Scatter plots showing an inverse Spearman's correlation between NPLOC4 expression and immune checkpoints from the TCGA database. (E) Scatter plots showing an inverse Spearman's correlation between NPLOC4 expression and infiltration of macrophages calculated by the TIMER algorithm from the TCGA database.(F) The overall survival curves of high and low NPLOC4 or macrophages groups in the CHCC-HBV (n=159) dataset were compared.

Figure S2

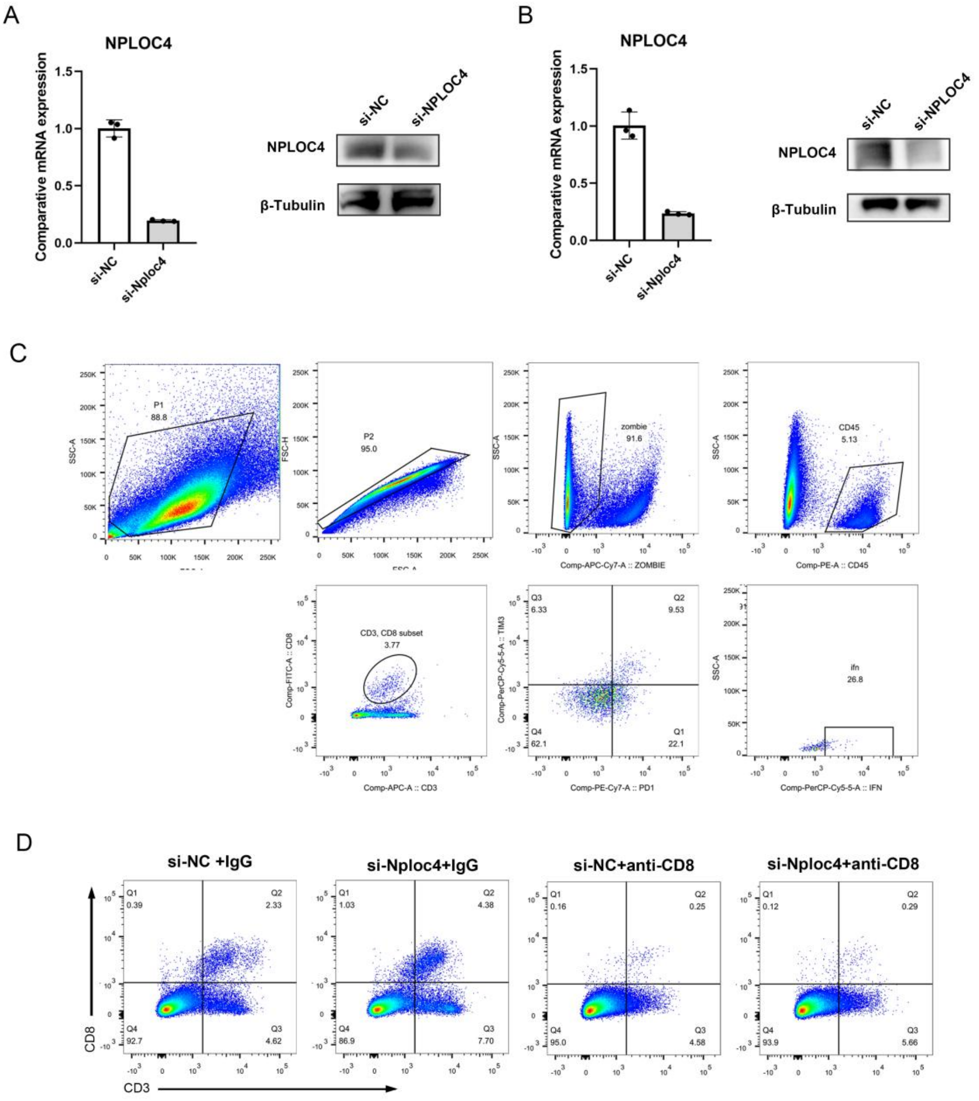

Figure S2. NPLOC4$^+$ TAMs mediate hepatocellular carcinoma progression by inhibiting CD8+ T cells infiltration and cytotoxic function. (A-B) Inhibition efficiency of NPLOC4 in BMDMs (A) and THP-1 (B) cells through Western blot analysis and qPCR. (C) Schematic diagram of logical door frame selection of CD8$^+$ T cells was listed, including exhausted status markers (marked as expression of PD-1, TIM-3) and cytotoxic function markers (marked as expression of IFN-γ). (D) CD8$^+$ T cell percentage in CD45$^+$ after anti-CD8 antibody treatment in subcutaneous tumor in NPLOC4 knockdown group. n: number of biological replicates. *$p < 0.05$; **$p < 0.01$; ***$p < 0.001$.

Figure S3

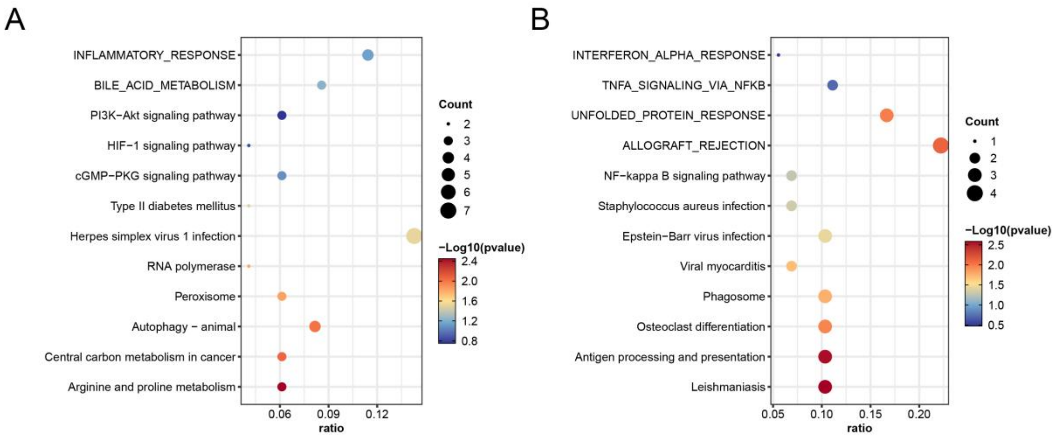

Figure S3. NPLOC4 knockdown reprograms TAMs by promoting polarization of antitumoral macrophages. (A) Pathway enrichment analysis of upregulated genes in NPLOC4^{+} TAMs. (B) Pathway enrichment analysis of downregulated genes in NPLOC4^{+} TAMs.

Figure S4

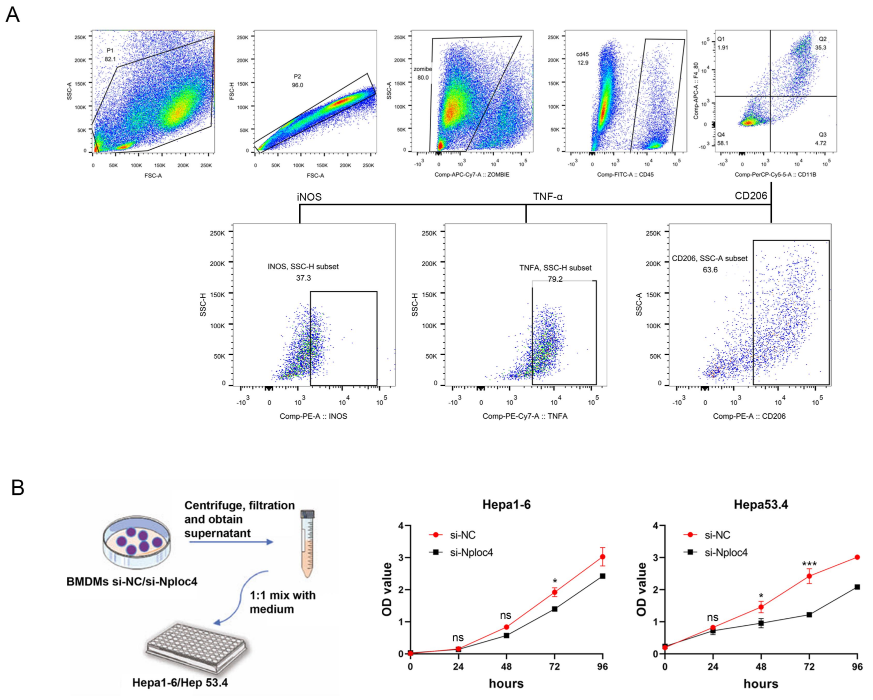

Figure S4. NPLOC4 knockdown reprograms TAMs by promoting polarization of antitumoral macrophages. (A) Schematic diagram of logical door frame selection of macrophages was listed, including M1 polarization (marked as expression of iNos, TNF-α) and M2 polarization (marked as expression of CD206). (B) The viability of Hepa1-6 and HEP53.4 cells after co-cultured with NPLOC4 knockdown macrophages detected by CCK-8 assay.

Figure S5

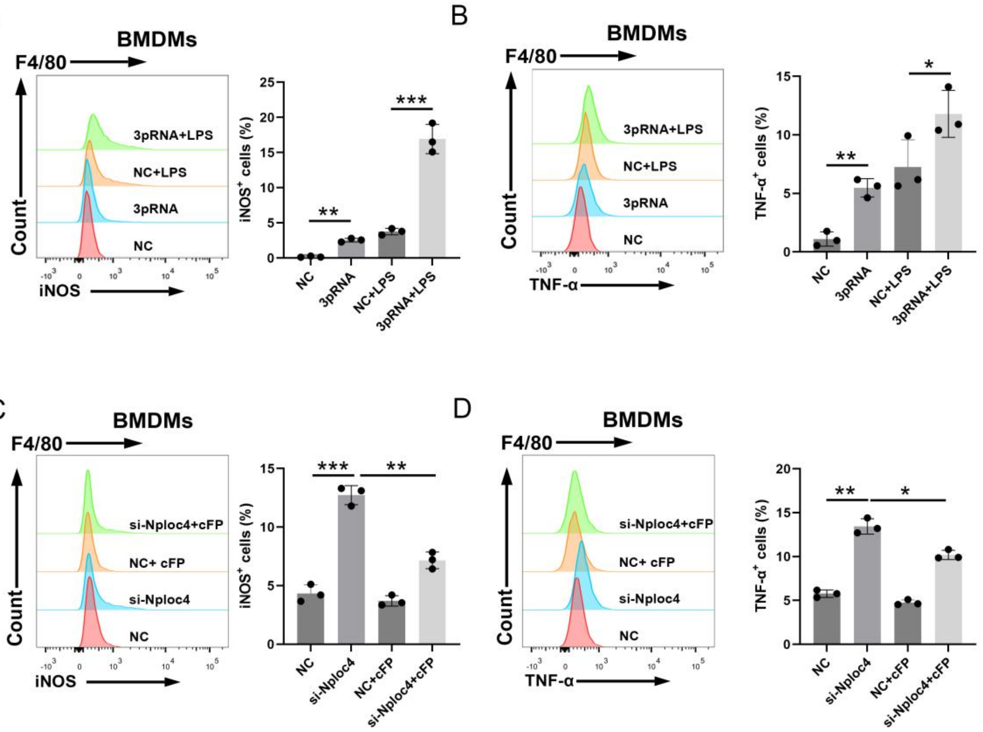

Figure S5. NPLOC4 mediate IFN-α inhibition by facilitating proteasomal degradation of RIG-I. (A-B) Representative histograms and percentages of $iNOS^+$ (A) and TNF-α^+ (B) in BMDM cells treated with 3pRNA. (C-D) Representative histograms and percentages of $iNOS^+$ (C) and TNF-α^+ (D) in si-NC or si-Nploc4 BMDM cells treated with cFP.

Figure S6

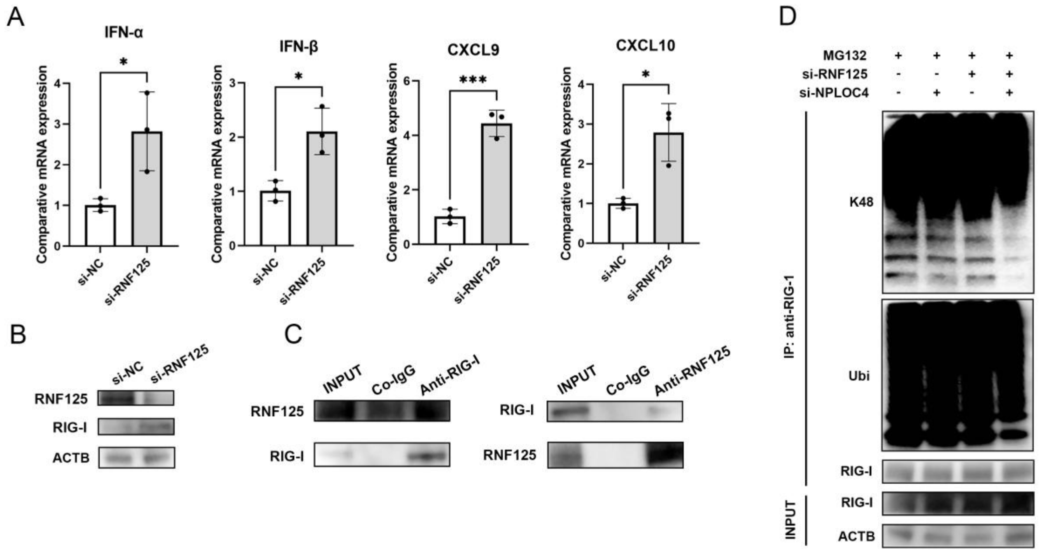

Figure S6. RNF125 is essential for NPLOC4 inhibition of RIG-I signaling. (A) qPCR showing mRNA expression of type I interferon related genes in THP-1 cells transfected with si-RNF125. (B) Immunoblot analysis of RIG-I of THP-1 cells transfected with si-NC or si-RNF125. (C) Immunoprecipitation assay for the interaction between RNF125 and RIG-I proteins in THP-1 cells. (D) Ubiquitination of RIG-I in THP-1 cells transfected with si-NC, si-RNF125 and si-NPLOC4.

Supplement Table 1

Clinical characteristic summary of patients with HCC

Variables	Total (n = 32)
Age (year)	53.69 ± 6.91
Gender	
Female	3 (9.38)
Male	29 (90.62)
Tumor differentiation grading	
G1	2 (6.25)
G2	20 (62.50)
G3	8 (25.00)
G4	2 (6.25)
Pathological grade (TNM)	
I	19 (59.38)
II	8 (25.00)
III	5 (15.62)
AFP level	
≤20 ng/mL	17 (53.12)
>20 ng/mL	15 (46.88)

Supplement Table 2

The target sequences used in the siRNA

Gene	Species	5'-3'
NPLOC4 siRNA	Human	GGTACCAGGTGTCCAATCA
Nploc4 siRNA	Mouse	GCCACCATTGAGCAGTTAT
RNF125 siRNA	Human	CUACCAGUCUAAAGAACAATT

Supplement Table 3

Primer information

Gene	Species		Sequence
NPLOC4	Human	Forward	GCCGAGACCCACAGCTATG
		Reverse	AGATAGTCCTCATCGAATGGCT
ACTB	Human	Forward	CATGTACGTTGCTATCCAGGC
		Reverse	CTCCTTAATGTCACGCACGAT
GAPDH	Human	Forward	GGAGCGAGATCCCTCCAAAAT
		Reverse	GGCTGTTGTCATACTTCTCATGG
IFNA1	Human	Forward	GCCTCGCCCTTTGCTTTACT
		Reverse	CTGTGGGTCTCAGGGAGATCA
IFNB1	Human	Forward	ATGACCAACAAGTGTCTCCTCC
		Reverse	GGAATCCAAGCAAGTTGTAGCTC
CXCL9	Human	Forward	CCAGTAGTGAGAAAGGGTCGC
		Reverse	AGGGCTTGGGGCAAATTGTT
CXCL10	Human	Forward	GTGGCATTCAAGGAGTACCTC
		Reverse	TGATGGCCTTCGATTCTGGATT
NOS2	Human	Forward	TTCAGTATCACAACCTCAGCAAG
		Reverse	TGGACCTGCAAGTTAAAATCCC
IL1B	Human	Forward	ATGATGGCTTATTACAGTGGCAA
		Reverse	GTCGGAGATTCGTAGCTGGA
ARG1	Human	Forward	GTGGAAACTTGCATGGACAAC
		Reverse	AATCCTGGCACATCGGGAATC
IL10	Human	Forward	GACTTTAAGGGTTACCTGGGTTG
		Reverse	TCACATGCGCCTTGATGTCTG
RIG-I	Human	Forward	CTGGACCCTACCTACATCCTG
		Reverse	GGCATCCAAAAAGCCACGG
Nploc4	Mouse	Forward	TCAAGCATGGCGATTTGTTGT
		Reverse	CGAGGTCGATGTTTCCATTTCAG
Actb	Mouse	Forward	CATCCACGAAACTACCTTCAACTCC
		Reverse	GAGCCGCCGATCCACACG
Gapdh	Mouse	Forward	AGGTCGGTGTGAACGGATTTG
		Reverse	TGTAGACCATGTAGTTGAGGTCA
Ifn-α	Mouse	Forward	CTACTGGCCAACCTGCTCTC
		Reverse	CCTTCTTGATCTGCTGGGCA
Ifn-β	Mouse	Forward	CAGCTCCAAGAAAGGACGAAC
		Reverse	GGCAGTGTAACTCTTCTGCAT
Cxcl9	Mouse	Forward	TCCTTTTGGGCATCATCTTCC
		Reverse	TTTGTAGTGGATCGTGCCTCG
Cxcl10	Mouse	Forward	CCAAGTGCTGCCGTCATTTTC
		Reverse	GGCTCGCAGGGATGATTTCAA

Nos2	Mouse	Forward	GTTCTCAGCCCAACAATACAAGA
		Reverse	GTGGACGGGTCGATGTCAC
Il1b	Mouse	Forward	GAAATGCCACCTTTTGACAGTG
		Reverse	TGGATGCTCTCATCAGGACAG
Arg1	Mouse	Forward	CTCCAAGCCAAAGTCCTTAGAG
		Reverse	GGAGCTGTCATTAGGGACATCA
Il10	Mouse	Forward	CTTACTGACTGGCATGAGGATCA
		Reverse	GCAGCTCTAGGAGCATGTGG
